# High-density lipoprotein suppresses tumor necrosis factor alpha production by mycobacteria-infected human macrophages

**DOI:** 10.1038/s41598-018-24233-1

**Published:** 2018-04-30

**Authors:** Manabu Inoue, Mamiko Niki, Yuriko Ozeki, Sachiyo Nagi, Evans Asena Chadeka, Takehiro Yamaguchi, Mayuko Osada-Oka, Kenji Ono, Tetsuya Oda, Faith Mwende, Yukihiro Kaneko, Makoto Matsumoto, Satoshi Kaneko, Yoshio Ichinose, Sammy M. Njenga, Shinjiro Hamano, Sohkichi Matsumoto

**Affiliations:** 1 Department of Bacteriology and Virology, Osaka-City University Graduate School of Medicine, Osaka, Japan; 2 Department of Bacteriology, Niigata University School of Medicine, Niigata, Japan; 3 Department of Parasitology, Institute of Tropical Medicine (NEKKEN) and the Graduate School of Biomedical Science, Nagasaki University, Nagasaki, Japan; 4 Food Hygiene and Environmental Health, Division of Applied Life Science, Graduate School of Life and Environmental Sciences, Kyoto Prefectural University, Kyoto, Japan; 5 Microbiological Research Institute, Otsuka Pharmaceutical, Tokushima, Japan; 6 Eastern and Southern Africa Centre of International Parasite Control (ESACIPAC), Kenya Medical Research Institute, Nairobi, Kenya; 7 Department of Eco-Epidemiology, Institute of Tropical Medicine (NEKKEN), Nagasaki University, Nagasaki, Japan; 8 Nagasaki University Institute of Tropical Medicine(NUITM)-Kenya Medical Research Institute (KEMRI), Nairobi, Kenya; 9 Department of Bacteriology, Institute of Tropical Medicine (NEKKEN), Nagasaki University, Nagasaki, Japan

## Abstract

Immune responses to parasitic pathogens are affected by the host physiological condition. High-density lipoprotein (HDL) and low-density lipoprotein (LDL) are transporters of lipids between the liver and peripheral tissues, and modulate pro-inflammatory immune responses. Pathogenic mycobacteria are parasitic intracellular bacteria that can survive within macrophages for a long period. Macrophage function is thus key for host defense against mycobacteria. These basic facts suggest possible effects of HDL and LDL on mycobacterial diseases, which have not been elucidated so far. In this study, we found that HDL and not LDL enhanced mycobacterial infections in human macrophages. Nevertheless, we observed that HDL remarkably suppressed production of tumor necrosis factor alpha (TNF-α) upon mycobacterial infections. TNF-α is a critical host-protective cytokine against mycobacterial diseases. We proved that toll-like receptor (TLR)-2 is responsible for TNF-α production by human macrophages infected with mycobacteria. Subsequent analysis showed that HDL downregulates TLR2 expression and suppresses its intracellular signaling pathways. This report demonstrates for the first time the substantial action of HDL in mycobacterial infections to human macrophages.

## Introduction

Low-density lipoprotein (LDL)-cholesterol (LDL-C) transfers cholesterol from the liver to peripheral tissues. However, a high level of LDL-C is a risk factor for cardiovascular diseases^1,2^ because it initiates atherosclerosis, leading to peripheral inflammations including the production of inflammatory cytokines and accumulation of macrophages and activated T cells. High-density lipoprotein (HDL), on the other hand, inversely transfers cholesterol from peripheral tissues to the liver. Indeed, high levels of serum HDL-cholesterol is correlated with a reduced risk of atherosclerosis and cardiovascular diseases^3–6^. When macrophages are filled with cholesterol, they become foam cells, which trigger massive inflammation during atherosclerosis. HDL removes cholesterol from macrophages through lipid transporter proteins, such as, ABCA1^7,8^, ABCG1^8,9^ and SR-B1^10^. This is considered as a part of the mechanism of anti-inflammatory effects by HDL. However, accumulated evidence also suggests a direct role of HDL in the suppression of inflammation^11–15^. It is thus likely that LDL and HDL have impacts on human health status concomitant with inflammation, such as, in infectious diseases.

Mycobacterial infections are still a serious threat to human health, especially *Mycobacterium tuberculosis* complex, an etiologic agent of tuberculosis (TB), which is responsible for the highest mortality among all single pathogens. The World Health Organization (WHO) estimated that 10.4 million people newly developed TB and 1.7 million people died from this disease in 2015, as indicated in the most recent report (WHO; Global Tuberculosis Report, 2017).

*M*. *tuberculosis* is an intracellular pathogen that is well adapted to ensure its survival in macrophages. Therefore, the function of macrophages and the pro-inflammatory cytokines that activate them are critical for the host defense^16^. A key pro-inflammatory cytokine, tumor necrosis factor alpha (TNF-α), activates macrophages and is essential for granuloma formation. A granuloma is the hallmark of mycobacterial infections^17^. It is a roundish immunopathological structure made up of activated macrophages, which prevent the dissemination of mycobacteria. The significant role of TNF-α in granuloma formation and hence TB control in humans was proven with the administration of TNF-α-neutralizing therapy, which disrupted TB granuloma and increased TB reactivation^18^.

Thus, activated macrophages participate in the prevention of TB progression. However, *M*. *tuberculosis* can persist without complete sterilization, accounting for the huge TB reservoir^19–21^. During persistent infections, *M*. *tuberculosis* uses cholesterol as a carbon source^22–26^. Host cholesterol is also essential for phagocytosis of mycobacteria by macrophages^27^. However, the immunomodulatory effects of cholesterol transporters, LDL and HDL, on mycobacterial diseases remain to be elucidated. In this study, we assessed the action of LDL and HDL on mycobacteria-infected human macrophages.

## Results

### Mycobacteria-infected human macrophages produce a large amount of TNF-α, which is suppressed by HDL

We differentiated the THP1 human acute monocytic cell line to macrophages by using phorbol 12-myristate 13-acetate (PMA) (THP1 macrophages). Macrophages were then cultured with or without adding varying doses of HDL or LDL (5–50 µg/ml) for 24 hours. We confirmed no significant differences in the viability rates of cells by addition of 5 to 50 µg/ml HDL or LDL, based on the results of the trypan blue-exclusion assays or assessment of cytoplasmic lactate dehydrogenase (LDH) enzyme activity in the culture medium.

To assess the effects of human plasma-derived HDL and LDL on mycobacteria infection of human macrophages, THP1 macrophages were infected with *M*. *tuberculosis* complexes, such as *Mycobacterium bovis* BCG (BCG) or *M*. *tuberculosis* H37Rv. Twenty-four hours after infection, we assessed inflammatory responses of the macrophages by measuring the levels of cytokines, including granulocyte/macrophage colony-stimulating factor (GM-CSF), IFN-γ, interleukin (IL)-2, IL-4, IL-6, IL-8, IL-10 and TNF-α, secreted in the culture supernatant.

Upon infection with BCG, we found a robust production of TNF-α (14820.02 ± 165.82 pg/ml), whose level was remarkably reduced with HDL treatment (Supplementary Fig. S1d). We found insignificant levels of IL-6 (76.64 ± 15.797 pg/ml) and IL-10 (40.03 ± 5.404 pg/ml) production (Supplementary Fig. S1a and b) and moderate levels of IFN-γ production (287.266 ± 4.738 pg/ml) (Supplementary Fig. S1c), all of whose expression levels were suppressed with HDL treatment. However, LDL suppressed the production of TNF-α and IFN-γ, but its effect was lower than that of HDL treatment, and it did not suppress the production of IL-6 or IL-10 (Supplementary Fig. S1a and b). The levels of other tested cytokines, such as IL-4, GM-CSF, IL-2 or IL-8, were below the detection limits in all samples.

**Figure 1:**
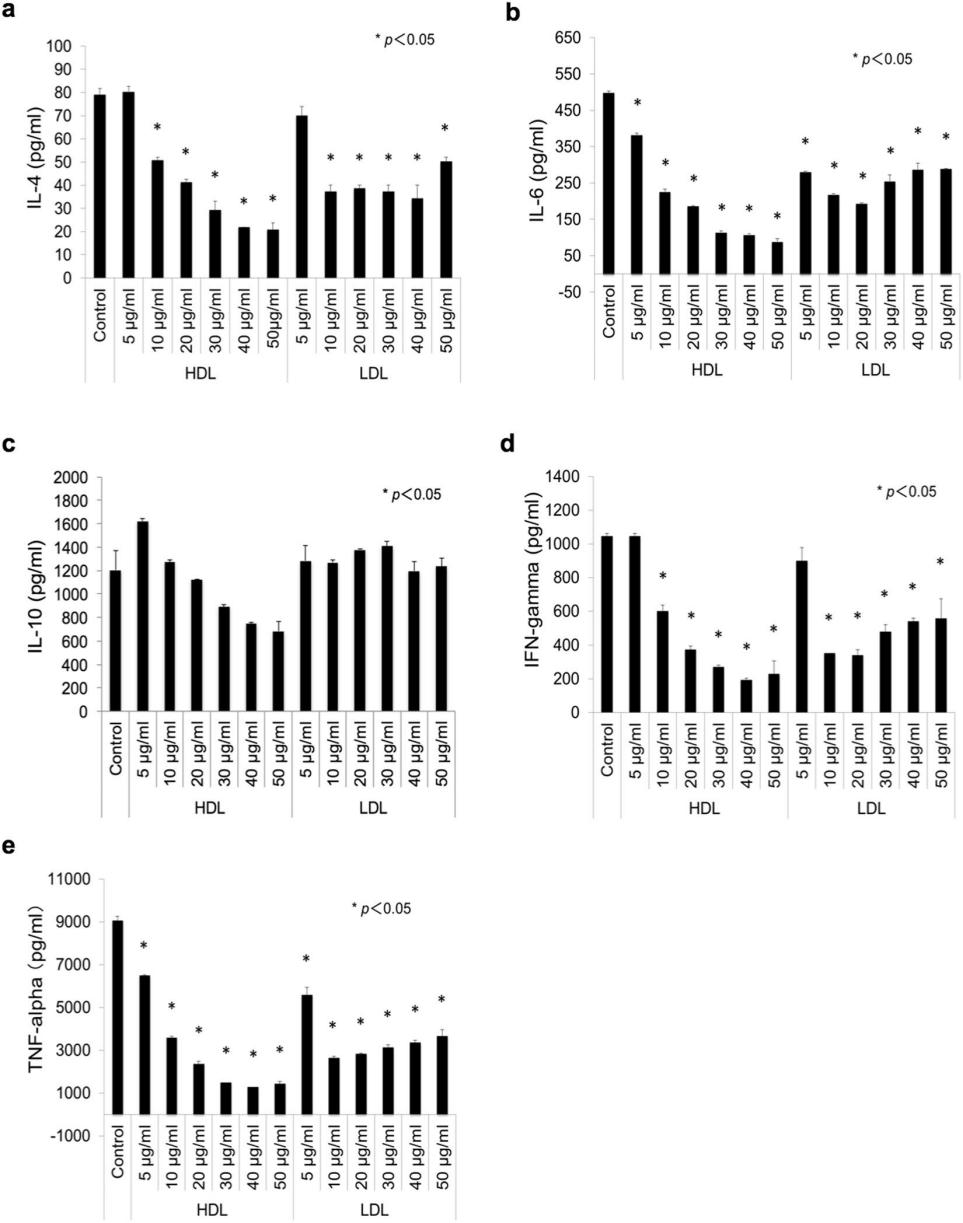
Effects of HDL and LDL on the expression of pro-inflammatory cytokines by *M*. *tuberculosis*-infected THP1macrophages. THP1-macrophages were cultured with or without adding HDL or LDL (5–50 µg/ml) for 24 hours. The treated macrophages were then infected with *M*. *tuberculosis* (multiplicity of infection = 10) for 24 hours (n = 4). The amounts of IL-4 (**a**), IL-6 (**b**), IL-10 (**c**), IFN-gamma (**d**), and TNF-α (**e**) from macrophages were measured using the Bio-Plex Multiplex System. ANOVA was used for test the differences in means.

Similar to infection with BCG, we found a high level of TNF-α production upon *M*. *tuberculosis* infection (9057.91 ± 219.07 pg/ml, Fig. 1e). We also found moderate levels of IL-6 (498.01 ± 4.78 pg/ml), IL-10 (1203.29 ± 168 pg/ml) and IFN-γ (1046.18 ± 16.83 pg/ml) production, and an insignificant level of IL-4 production (79.16 ± 2.6 pg/ml) (Fig. 1a–d). Notably, treatment with HDL significantly reduced the production of all of these cytokines in a dose-dependent manner, except for IL-10. LDL treatment reduced the levels of IL-4, IL-6, IFN-γ and TNF-α production, but to a lesser extent than HDL treatment, and these changes did not show dose-responsiveness. The levels of other tested cytokines, such as GM-CSF, IL-2 and IL-8, were below the detection limits in all samples. These data suggest that among the tested cytokines, TNF-α is the most abundantly produced upon infection of THP1 macrophages with *M*. *tuberculosis* complex and HDL suppresses its production in a dose-dependent manner.

We also assessed the effects of HDL on *M*. *tuberculosis*-infected human macrophages that were differentiated from CD14 positive monocytes derived from peripheral blood mononuclear cells (PBMC). As observed in THP1 macrophages, HDL but not LDL treatment of the *M*. *tuberculosis*-infected macrophages significantly suppressed TNF-α production by PBMC-derived human macrophages (Supplementary Fig. S2). This shows the common responses to HDL by both THP1 macrophages and PBMC-derived ones.

### HDL but not LDL promotes entry of both BCG and *M*. *tuberculosis* into THP1 macrophages

HDL functions as a remover of cellular cholesterol in the periphery. In addition, previous reports showed that the removal of cholesterol suppressed BCG-infection of mouse macrophages^27^. This suggests the possibility that HDL inhibits phagocytosis of mycobacteria and in turn reduces infection-dependent TNF-α production. To investigate this possibility, we determined the infecting number of mycobacteria by counting colony forming units (CFUs) 24 hours after infection of THP1 macrophages. The data revealed that CFUs of BCG and *M*. *tuberculosis* in LDL-treated macrophages were similar to those in control macrophages. In contrast, HDL-treated macrophages harbored a significantly higher number of both BCG and *M*. *tuberculosis* (Supplementary Fig. S3). These data suggest that HDL enhances mycobacterial infections of macrophages contrary to prior expectations.

### HDL inhibits the transcription of TNF-α and TNF receptors

**Figure 2:**
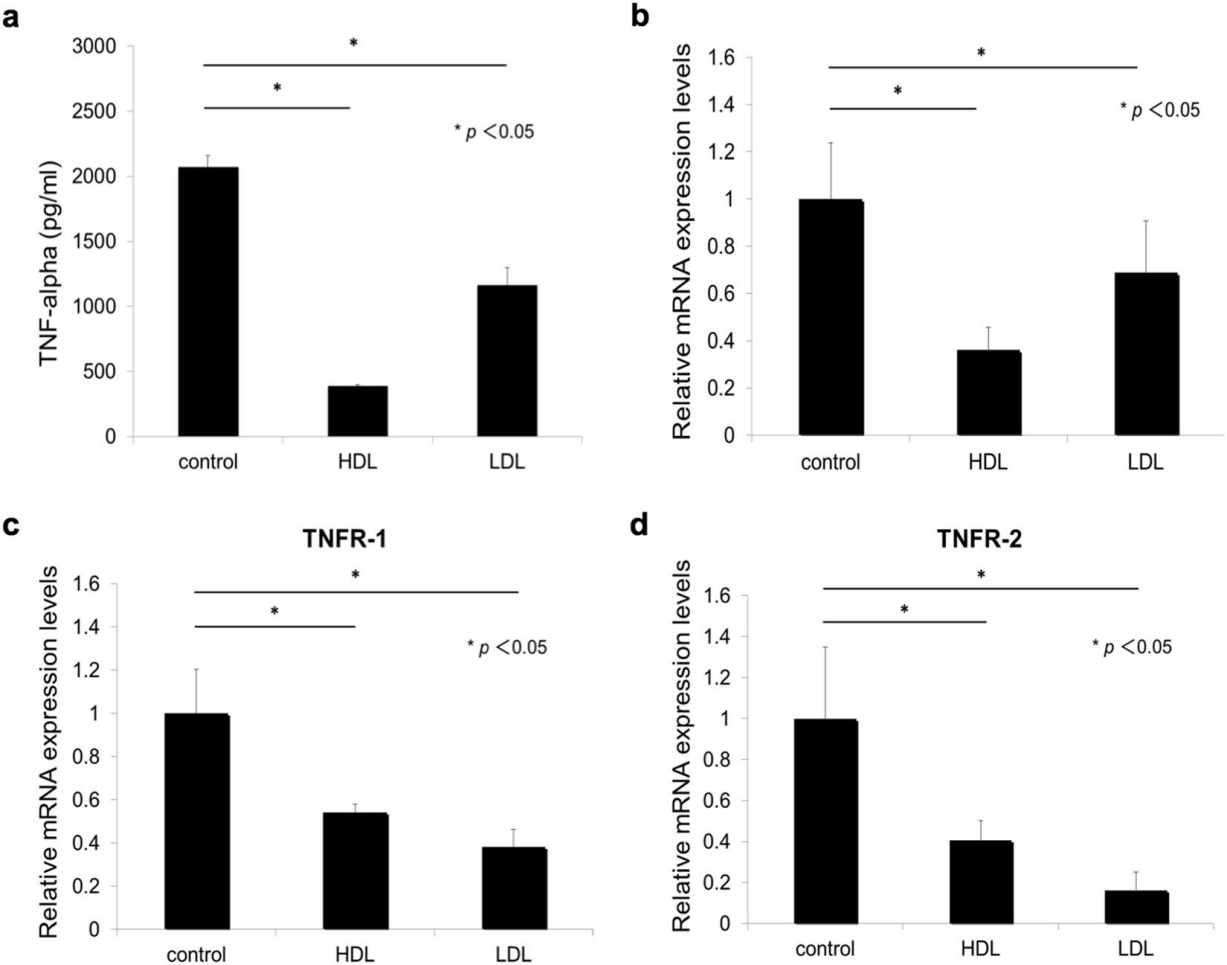
Effect of HDL on the expression of TNF-α and its receptors in BCG-infected macrophages. (**a**) THP1 macrophages were cultured with or without adding HDL or LDL (50 µg/ml) for 24 hours. The treated macrophages were then infected with BCG (multiplicity of infection = 10) for 24 hours. The amounts of TNF-α in the cell culture supernatants were measured by ELISA. This assay was repeated three times. (**b**–**d**) THP1 macrophages were cultured with or without adding HDL or LDL (50 µg/ml) for 24 hours and were then infected with BCG (multiplicity of infection = 10) for 6 hours. TNF-α (**b**), TNFR-1 (**c**), and TNFR-2 (**d**) mRNA expression levels were quantified using real-time PCR (n = 3) and normalized to GAPDH expression. ANOVA was used to test for the differences of in means.

Despite elevated mycobacterial entry into THP1 macrophage, the production of pro-inflammatory cytokines is inhibited by addition of HDL, suggesting some unknown systematic mechanism of HDL to this effect. We next analyzed whether the regulation occurs at the transcriptional level. In this experiment, the amount of TNF-α from BCG-infected THP1 macrophages in the control group, the group treated with 50 µg/mL HDL or LDL was 2067.2 pg/ml, 388.2 pg/ml, and 1161.3 pg/ml, respectively (Fig. 2a). The mRNA levels of TNF-α in BCG-infected THP1 macrophages pretreated with HDL were suppressed by 64% (Fig. 2b), suggesting the presence of transcriptional regulation.

Prior work indicated that TNF-α transcription is regulated by autocrine systems through the activation of the NF-κB pathway by the bonding of TNF-α with TNF receptors (TNFR-1 and TNFR-2)^28^. We evaluated the effects of HDL on TNFR-1 and TNFR-2 mRNA expression levels. We found that these were both significantly suppressed by pretreatment with HDL (Fig. 2c and d), but to a lesser extent compared with LDL-treatment. This suggested the presence of a HDL-specific mechanism other than down-regulation of TNFRs.

### HDL suppresses TLR2-dependent production of TNF-α by THP1 macrophages

A recent study showed that, in mouse bone marrow-derived macrophages, HDL induces transcription factor 3 (ATF3), which in turn suppresses the transcription of the genes encoding pro-inflammatory cytokines^12^. We, therefore, assessed the upregulation of ATF3 in BCG-infected THP1 macrophages. However, we could not find any significant increase in ATF3 with HDL-treatment compared with the untreated control and with LDL treatment (Supplementary Fig. S4).

**Figure 3:**
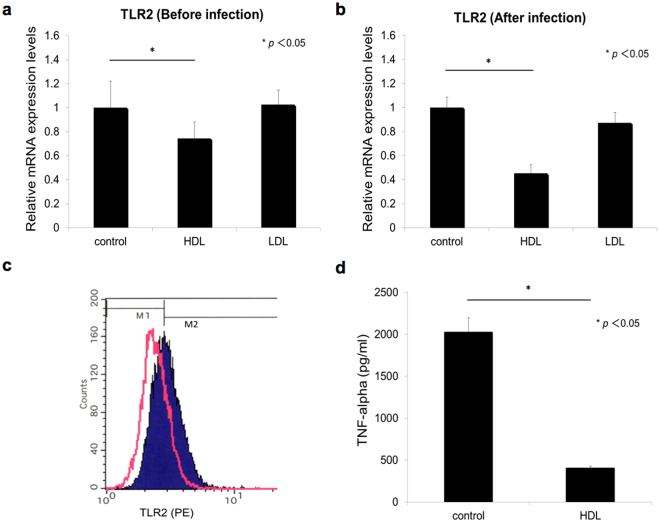
HDL suppresses TLR2 expression of THP1 macrophages. (**a**) Macrophages were cultured with or without adding HDL or LDL (50 µg/ml) for 6 hours. The TLR2 mRNA expression levels were then quantified using real-time PCR (n = 3) and normalized to GAPDH expression. (**b**) Macrophages were cultured with or without adding HDL or LDL (50 µg/ml) for 24 hours and then infected with BCG (multiplicity of infection = 10) for 6 hours. The TLR2 mRNA expression level was quantified by real-time PCR (n = 3). ANOVA was used to test for the differences of in means. These assays were repeated twice. (**c**) THP1macrophages were cultured with (merged with pink) or without (filled with purple) addition of HDL (50 µg/ml) for 6 hours. Surface TLR2 was detected with PE-labeled anti-TLR2 antibody and analyzed by FACScan. The border of M1 and M2 was set at the peak of untreated THP1 cells stained by TLR2 antibody. Calculated % of cells in the M2 area of control cells and HDL-treated cells were 55.8% and 22.4%, respectively. (**d**) THP1 macrophages were cultured with or without addition of HDL (50 µg/ml) for 24 hours and were then stimulated with the TLR2 ligand Pam3CSK4 (50 ng/ml) for 24 hours. The amounts of TNF-α in the cell culture supernatants (n = 2) were measured by ELISA. ANOVA was used to test for the differences of in means. This assay was repeated twice.

Stimulation of pattern recognition receptors, such as TLRs, is the predominant trigger of TNF-α transcription in mycobacteria-infected macrophages. TLR1/2, 2/6, 4 and 9 are involved in the recognition of mycobacterial components, and TLR2 is considered to be the most responsible receptor for induction of TNF-α^29–32^. We analyzed the effect of HDL on the mRNA levels of TLR2. In uninfected macrophages, HDL reduced the TLR2 mRNA level by around 80% compared with that in untreated or LDL-treated ones (Fig. 3a). Importantly, HDL treatment reduced the TLR2 mRNA level to around 40% in mycobacteria-infected macrophages compared with the untreated control (Fig. 3b). Flow cytometry data analysis showed that the level of surface TLR2 of BCG-infected THP1 macrophages was reduced from 55.8% to 22.4% by the treatment with HDL as shown in Fig. 3c.

We next assessed whether TLR2-dependent TNF-α production was impaired by HDL. We found that, indeed, HDL reduced TNF-α production by macrophages stimulated with 50 ng/ml of PAM3CSK4, a ligand for the TLR2/TLR1 homodimer (Fig. 3d).

**Figure 4:**
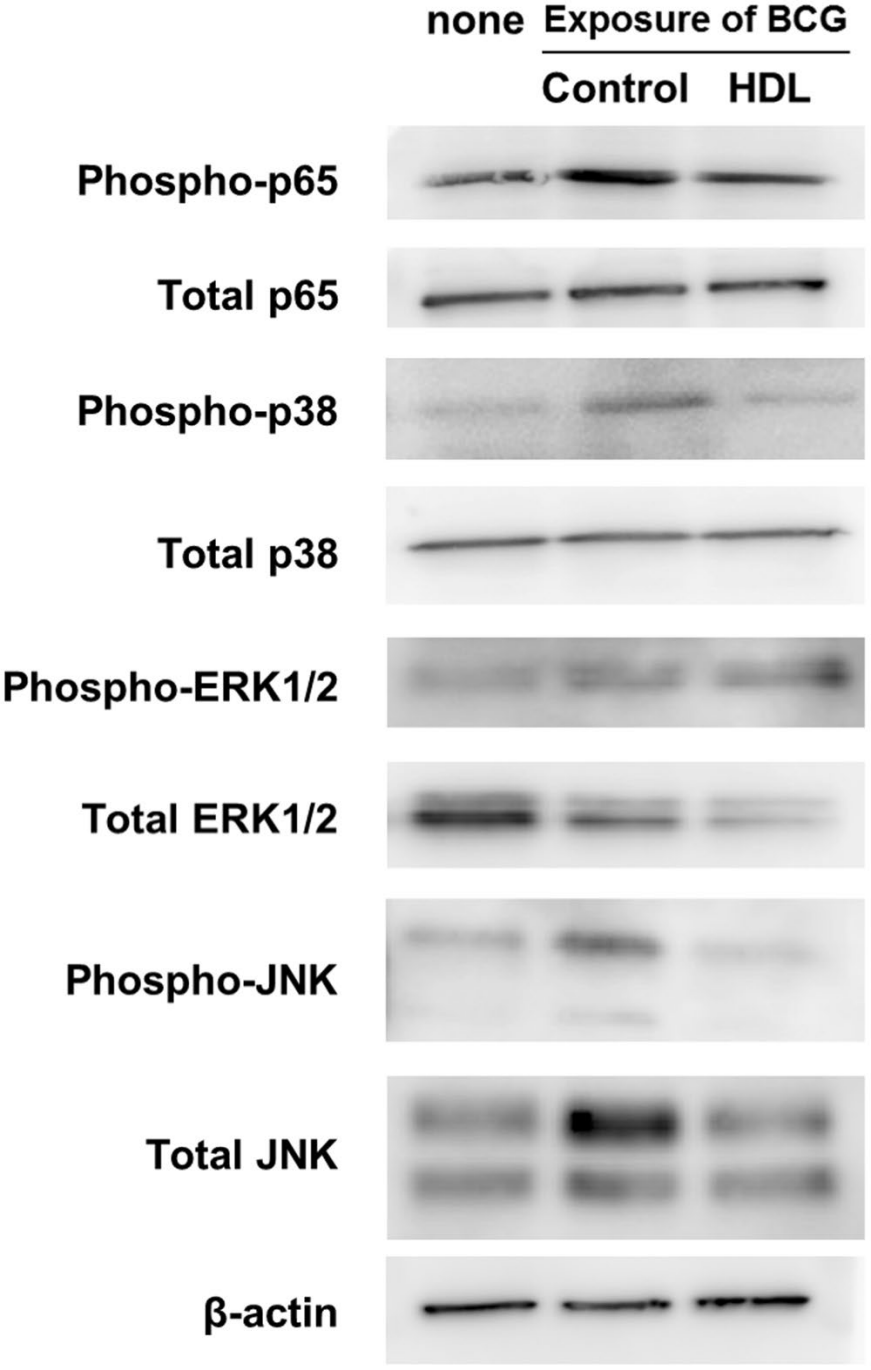
HDL impairs activation of TLR2-mediated intracellular signalings. THP1 macrophages were cultured with or without (control) adding HDL (50 µg/ml) for 24 hours. The macrophages were then infected with BCG (multiplicity of infection = 10) for 15 minutes. Immunoblot of p65 phosphorylation (phospho-p65), total p65, p38 phosphorylation (phospho-p38), total p38, ERK phosphorylation (phospho-ERK), total ERK, JNK phosphorylation (phospho-JNK) and total JNK (relative to total β-actin) were detected. Each whole cell lysate was fractionated by SDS-PAGE and transferred on a membrane.Immunoblot experiments were then performed to validate the level of each target protein. The western blot bands of phospho-p65 (65 kDa) total p65 (65 kDa), phospho-p38 (43 kDa), total p38 (43 kDa), phospho-ERK (44 and 42 kDa), total ERK (44 and 42 kDa), phospho-JNK (46 and 54 kDa) and total JNK(46 and 54 kDa) and total beta-actin (43 kDa)are displayed in the figure. Wholemembranes were also displayed in Supplemental Fig. S5. The immunoblots are representative of three independent experiments.

To confirm that TLR2-mediated signaling pathways were impaired by HDL, we analyzed the activation (phosphorylation) of NF-κB (p65) and p38, ERK and JNK mitogen-activated protein kinases (MAPKs) by immunoblot of the cell lysates derived from HDL-treated BCG-infected macrophages. The data indicated 29%, 38% and 45% reduced phosphorylation of P65, P38 and JNK, respectively. This showed that HDL indeed suppressed activation of both major cellular signaling pathways for the transcription of the TNF-α gene^30,33–35^ (Fig. 4 and Supplementary Fig. S5). By contrast, HDL did not influence the phosphorylation of ERK.

### TLR2 is responsible for TNF-α production by THP1 macrophages upon infection with mycobacteria

**Figure 5:**
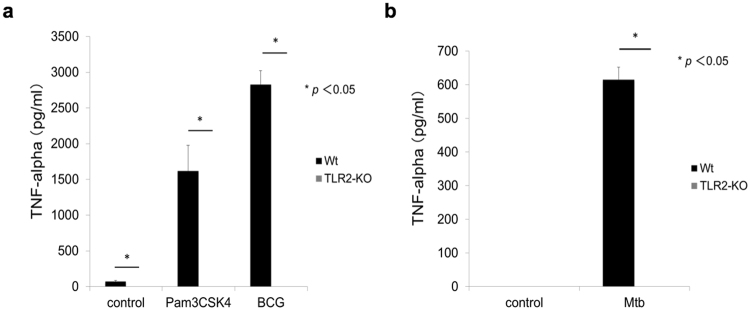
TLR2 is critical for TNF-α production from THP1 macrophages upon mycobacterial infection. (**a**) THP1 macrophages and its TLR2-KO cells were stimulated with the TLR2 ligand, Pam3CSK4 (50 ng/ml) or infected with BCG (multiplicity of infection = 10) for 24 hours, and the amount of TNF-α in the cell culture supernatant (n = 3) was measured by ELISA. (**b**) THP1 macrophages and TLR2-KO cells were infected *M*. *tuberculosis* (multiplicity of infection = 10) for 24 hours, and the amount of TNF-α in the cell culture supernatant (n = 3) was measured by ELISA. ANOVA was used to test for the differences of in means.

TLR2 is a receptor responsible for TNF-α production upon mycobacterial infection of mouse macrophages^36,37^. While its similar role in human macrophages has been implied^38^, its exact responsibility has not been elucidated^36–38^. To establish its exact role, we generated TLR-2 knock-out (KO) THP1 cells using the CRISPR-Cas9 system (Supplementary Fig. S6). As expected, the TLR-2 KO THP1 macrophage could not produce TNF-α when stimulated with PAM3CSK4. In this experimental setting, we observed that TNF-α production was impeded because of TLR2-deficiency upon BCG (Fig. 5a) and *M*. *tuberculosis* (Fig. 5b) infection. This demonstrated the critical role of TLR2 in TNF-α production by mycobacteria-infected human macrophages.

## Discussion

This study demonstrates for the first time that HDL dose-dependently suppresses the production of pro-inflammatory cytokines, such as, IL-6, TNF-α and IFN-γ by THP1 macrophages infected with *M*. *tuberculosis* (Fig. 1). The same was true when macrophages differentiated from CD14^+^ monocytes derived from human blood were used (Supplementary Fig. S2). LDL treatment also reduced the production of cytokines but its effect was lower than that of HDL and it did not show dose responsiveness (Fig. 1), suggesting its minor function or non-functional artifacts. TNF-α was the most abundantly produced cytokine among those tested. It plays a key role in the immune defense during both active and latent TB^39–44^. IL-6 and IFN-γ also contribute to protection against mycobacterial diseases^40,45–48^. Inversely, however, massive production of these pro-inflammatory cytokines is harmful to the body. Our data thus suggest that HDL has the potential to modulate immune responses against mycobacterial infections.

HDL-dependent suppression of pro-inflammatory cytokine production was not owing to inhibition of mycobacterial infection of macrophages. Rather, HDL treatment enhanced these infections (Supplementary Fig. S3). In the presence of sera, mycobacteria enters macrophages mainly through the complement receptor^49^. The contribution of other receptors, such as mannose receptor and the scavenger receptor B1 (SR-B1) have been reported as well^29,50^. We investigated the expression of these receptors but could not find any upregulation in HDL-treated THP1 macrophages. It is thus considered that other uncharacterized receptors may contribute to this process. The other possibility relates to the dynamic quantitative change of the macrophage membrane fluidity as a result of a change in cholesterol levels that should affect phagocytosis^51^.

Recently ATF3 was proposed to be the master transcriptional regulator in HDL-dependent suppression of pro-inflammatory cytokines in mice bone marrow-derived macrophages^12^. However, we could not find any upregulation of ATF3 in THP1 macrophages treated with HDL (Supplementary Fig. S4). This discrepancy may be due to the difference in responses between mouse and human macrophages.

We investigated other possible molecular mechanisms to explain the HDL-dependent suppression of TNF-α production. TLR2 is a host-protective molecule against mycobacterial infections in both mouse and human^52–56^ and is considered a predominant receptor for TNF-α production. We thus next focused on TLR2^38^. We found that HDL suppressed TLR2 expression (Fig. 3), cellular response to the TLR2-specific ligand (Fig. 3d) and TLR2-related cellular signaling pathways (Fig. 4). We then proved that TLR2 was a key receptor for TNF-α production upon mycobacterial infection by creating a TLR2-KO THP1 cell (Fig. 5). Taken together these data indicate the significant role of TLR2 in TNF-α production upon mycobacterial infection and the involvement of down-regulation of TLR2 by HDL in the suppression of TNF-α production by mycobacteria-infected THP1 macrophages.

Previously, Tang *et al*. showed that apolipoprotein A-1, a component of HDL, interacts with ABCA1 and activates STAT3, which in turn suppresses the production of pro-inflammatory cytokines^57^. ABCA1 transcription was remained in HDL-treated THP1 macrophages, although it was significantly reduced upon LDL treatment (Fig. S7). This implies the possibility of interaction between down-regulation of TLR2 and activation of the JAK/STAT3 signaling pathway, which should be clarified in future studies.

There are few epidemiological cohort studies that estimate the relationship between HDL levels and tuberculosis. Two papers showed that decrease of HDL-cholesterol levels was correlated with severity of the disease in active TB patients^58^. Another study showed that the level of HDL-c, but not LDL-c or total cholesterol was significantly lower in 115 TB patients compared to 70 pneumonia patients and 30 healthy control^59^. In contrast, hypercholesterolemia impairs protective immunity to tuberculosis in mice model^60^. A recent adolescent cohort study with 6,363 enrolled participants identified the upregulation of apolipoprotein A-1 as a risk factor of TB progression from latent infection^61^. It is also known that *M*. *tuberculosis* uses a host cholesterol as a major carbon source during infection^22–26^, implying that higher cellular cholesterol levels may activate TB disease progression. Taken together, these suggest the important but controversial role of HDL in the determination of TB disease outcome. Further studies are in demand to clarify the effect of HDL and LDL in clinical situations of mycobacterial diseases including initial infection, asymptomatic states, and active diseases.

HDL is a highly condensed lipoprotein fraction in plasma. The most abundant apolipoprotein is A-I and the second one is apo A-II, but it is composed of over different 80 kinds of proteins and their variants. To identify which components and sub-fractions of HDL are responsible for their activity are next important issues to be clarified.

The downregulation of TLR2 by HDL interferes with the general recognition of pathogen-associated molecular patterns and is not limited to mycobacterial diseases. It is thus likely that HDL modulates host protective responses against other pathogens. While HDL is related to lower risk of vascular disorders contributing to human longevity, this study suggests the importance of taking into account the risk of immune suppression posed by HDL.

## Methods

### Isolation of HDL and LDL from healthy donors

HDL and LDL were isolated by a method previously developed^62^. Briefly, freshly drawn blood samples were collected (1 mM EDTA as anti-coagulant) and centrifuged at 2,000 × *g* for 15 min. Chylomicrons were then removed from plasma by additional centrifugation at 100,000 × *g* for 10 min. For each sample, four volumes of plasma were mixed with one volume of OptiPrep™ (Axis-Shield, Dundee, Scotland), and 2.8 ml of the resulting mixture was transferred to an OptiPrep™ tube. Solution B (0.85 g of NaCl dissolved in 50 ml water) was layered on top of this mixture, followed by a layer of 10 ml of 100-mM Hepes stock solution. After sealing the tubes, the samples were centrifuged at approximately 350,000 × *g* for 3 hours at 16 °C. The HDL and LDL fractions were collected by using disposable syringes with 23-gauge needles.

### Cells

THP1 cells, a human acute monocytic cell line that was purchased from the Human Science Foundation (Tokyo, Japan), were cultured in RPMI-1640 supplemented with 10% HI-FBS (Thermo Fisher Scientific, Waltham, MA, USA). Differentiation was achieved by re-suspending the cells at 8 × 10^5^ cells/ml in Dulbecco’s modified Eagle’s medium (Wako) supplemented with 10% heat-inactivated fetal bovine serum and penicillin-streptomycin with the addition of 100 nM phorbol 12-myristate 13-acetate (Sigma) for 48 hours. Non-adherent cells were removed by aspiration of the supernatant followed by replacement with fresh medium.

The human blood PBMC was obtained by using Optiprep (Axis-Shield, Dundee, Scotland) in accordance with the manufacturer’s instructions. CD14^+^ monocytes were then isolated from PBMC by the AutoMacs separation system using anti-CD14 magnetic microbeads (Miltenyi Biotec, Germany). The CD14^+^ human monocytes were differentiated to macrophages by commercially available macrophage differentiation kit (R&D systems, Minnesota).

### Reagents

*Salmonella*-derived LPS and Pam_3_CSK_4_ were purchased from Sigma and R&D Systems (Minneapolis, USA), respectively.

### Cell viability

We assessed the cell viability by using two methods: 1) trypan blue-exclusion assay; and 2) Cytotoxicity Detection Kit^plus^, which measures the LDH release from dead cells. Differentiated macrophages were cultured with or without adding HDL or LDL (5–50 µg/ml) for 24 hours. In trypan blue-exclusion assays, cells were stained with trypan blue solution, loaded into a hemocytometer and immediately examined under a microscope. When using the Cytotoxicity Detection Kits^plus^ (LDH), the cytoplasmic LDH enzyme activity in the culture medium was measured according to the manufacturer’s instructions.

### Measurement of mycobacterial infection by CFU assays

*In vitro* infection experiments using BCG and *M*. *tuberculosis* were conducted in BSL2 and BSL 3 level facilities in the Department of Bacteriology, Niigata University School of Medicine after approval by the Institutional Biosafety Committee of Niigata University. Differentiated macrophages were infected with *M*. *tuberculosis* strain H37Rv or BCG at a multiplicity of infection of 10 for 3 hours, washed, and lysed with PBS containing 0.5% Triton X-100. CFU assays were performed by harvesting serially diluted suspensions on Middlebrook 7H11 agar plates supplemented with oleic, albumin, dextrose, and catalase (OADC) at 37 °C for 3 weeks to quantify the number of mycobacteria that had infected the macrophages.

### Quantitative measurement of TNF-α, TNFR-1, TNFR-2, TLR2, and ATF3 mRNA expression levels in THP1 macrophages

Differentiated macrophages were harvested, and total RNA was extracted using TRIzol® (Thermo Fisher Scientific) according to the manufacturer’s instructions. The resulting RNA was then treated with RNase-free DNase (Ambion, California, USA) to remove any potential DNA contamination. After being reverse-transcribed to cDNA using the Super Script First Strand Synthesis System for reverse-transcription PCR (Invitrogen, Massachusetts, USA), triplicate cDNA aliquots were amplified with sequence-specific primers and SYBR green (Applied Biosystems by Life Technologies, California, USA) using 7500 Fast Real-Time PCR Systems (Applied Biosystems by Life Technologies). All reactions were repeated independently at least three times to ensure the reproducibility of the results. All primers were purchased from Sigma (Supplementary Table S1).

### Measurement of secreted TNF-α level by enzyme-linked immunoabsorbent assay (ELISA)

TNF-α levels in the cell-culture supernatant were measured using human TNF-α ELISA kits (R&D Systems) according to the manufacturer’s instructions.

### Measurement of secreted IL-4, IL-6, IL-10, IFN-γ and TNF-α level by the Bio-Plex Multiplex System

IL-4, IL-6, IL-10, IFN-γ and TNF-α levels in cell-culture supernatants were measured using the Bio-Plex Multiplex System (Bio-Rad, California, USA) according to the manufacturer’s instructions.

### Flow cytometry

Following treatment with HDL (50 µg/ml), cells were washed with ice-cold PBS and then fixed with 80% methanol for 5 min and washed again with ice-cold PBS. Cells were then incubated with unlabeled anti-Fc receptor antibody (eBioscience, San Diego, USA) in PBS in the dark at 4 °C for 20 min to block the Fc receptor. The cells were subsequently incubated with PE-labeled anti-human CD282 (TLR2) (clone: TL2.1) (eBioscience) in PBS at room temperature in the dark for 20 min. Cells were then washed twice with ice-cold PBS, resuspended in ice-cold PBS, and analyzed on a flow cytometer (FACSCalibur HG, Becton Dickinson). An isotype-matched antibody, mouse IhG2aκ Iso Control PE (clone: eBM2a) (eBioscience), of irrelevant specificity was used as a control. The control cells were analyzed using FACScan (BD Bioscience, Franklin Lakes, USA).

### Gene editing of TLR2 by CRISPR-Cas9 system

To generate TLR2-KO THP1 cells, the CRISPR-Cas9 system was applied. Niigata University Recombinant DNA Advisory Committee approved CRISPR/Cas9 gene knock out of TLR2 gene in THP1 cells in the BSL2 laboratory of Department of Bacteriology, Niigata University School of Medicine. The sequence of guide RNA (gRNA) synthesis, which targets the human TLR2 gene, was GACTGTACCCTTAATGGAGT(TGG). It was inserted into pSpCas9 (BB)-2A-Puro (PX459) V2.0 (Addgene, Cambridge, MA, USA) as previously described^63^. After insertion of the gRNA sequence, the pX459 vector was transfected into THP1 cells by electroporation with Gene Pulser Xcell (Bio-Rad, Hercules, CA, USA). For electroporation, THP1 cells were collected by centrifugation from culture and were suspended by the serum-free RPMI-1640 medium at 1 × 10^7^ cells/ml. In a 0.4-cm cuvette, 10 μg of plasmid vector and 0.4 ml of cell suspension were mixed and incubated for 10 min, and then pulsed once with 260 V and 1050 μF. After electroporation, cells were grown in RPMI-1640 medium supplemented with 10% of FBS. Forty-eight hours after electroporation, cells were selected by 1 μg of puromycin (Invivogen, San Diego, CA, USA) for 48 hours. To obtain a monoclonal cell population, cloning by limiting dilution was performed. Gene editing of TLR2 was confirmed by the PAGE-based genotyping protocol^64^ and by determining the DNA sequence of a targeted region of the TLR2 exon.

### Immunoblotting of p65, p38, ERK1/2 and JNK

More than 5 × 10^6^ differentiated THP1 macrophages were lysed with 1 Xlysis buffer containing 1% NP-40, 20 mM Tris-HCl pH 7.5, 2 mM EDTA and 150 mM NaCl, supplemented with a protease inhibitor cocktail (Nacalai Tesque) and 100 mM NaF, on ice for 10 min. The samples were then centrifuged at 16,000 g for 5 min at 4 °C, and the supernatant was analyzed by immunoblotting of whole cell lysates. A total of 5 µg of each lysate, of which the protein concentration was determined by Advanced Protein Assay (Cytoskeleton, Inc.), was run on 12.5% SDS-PAGE gel. After SDS-PAGE, fractionated proteins were transferred onto PVDF membranes (Millipore) and blocked in Tris-buffered saline (pH 7.5) containing 1% (wt/vol) skim milk and 0.05% Tween 20 before overnight incubation with each specific primary antibody. The following antibodies were purchased from Cell Signaling TECHNOLOGY and used in this study: anti-phospho-NF-κB p65 (Ser536) (93H1) Rabbit mAb (1:1000 dilution), anti-NF-κB p65 (D14E12) XP Rabbit mAb (1:1000 dilution), anti-phospho-p38 MAP Kinase (Thr180/Tyr182) antibody (1:1000 dilution), anti-p38 MAPK antibody (1:1000 dilution), anti-phospho-p44/42 MAPK (Erk1/2) (Thr202/Tyr204) (D13.14.4E) XP Rabbit mAb (1:2000 dilution), anti-p44/42 MAPK (Erk1/2) (137F5) Rabbit mAb (1:1000 dilution), anti-phospho-SAPK/JNK (Thr183/Tyr185) (81E11) Rabbit mAb (1:1000 dilution), anti-SAPK/JNK antibody (1:1000 dilution) and anti-β-Actin (C4): sc-47778 antibody (1:2500 dilution). The membranes were then washed and incubated with appropriate secondary antibodies, and the antibody reactions were detected with Immobilon™ western (Millipore).

### Data analysis

ANOVA was used to analyze *in vitro* assay data. All statistical analyses were carried out using SPSS (ver. 21, IBM, NY, USA), and *p*-values less than 0.05 were considered significant^65–69^.

## Electronic supplementary material

Supplemental Data
