# Supplementary material for: High-density lipoprotein suppresses tumor necrosis factor alpha production by mycobacteria-infected human macrophages
Source: Sci Rep. 2018 Apr 30;8:6736. doi: 10.1038/s41598-018-24233-1 (PMC5928146; doi:10.1038/s41598-018-24233-1)
Supplement: Supplementary file 1 — Supplemental Data [file 41598_2018_24233_MOESM1_ESM.docx]

**Supplementary Data**

**High-density lipoprotein suppresses tumor necrosis factor alpha production by mycobacteria-infected human macrophages**

Manabu Inoue, Mamiko Niki, Yuriko Ozeki, Sachiyo Nagi, Evans Asena Chadeka, Takehiro Yamaguchi, Mayuko Osada-Oka, Kenji Ono, Tetsuya Oda, Faith Mwende, Yukihiro Kaneko, Makoto Matsumoto, Satoshi Kaneko, Yoshio Ichinose, Sammy M Njenga, Shinjiro Hamano, and Sohkichi Matsumoto

**Supplementary Table S1. Oligonucleotides used in this study**

**
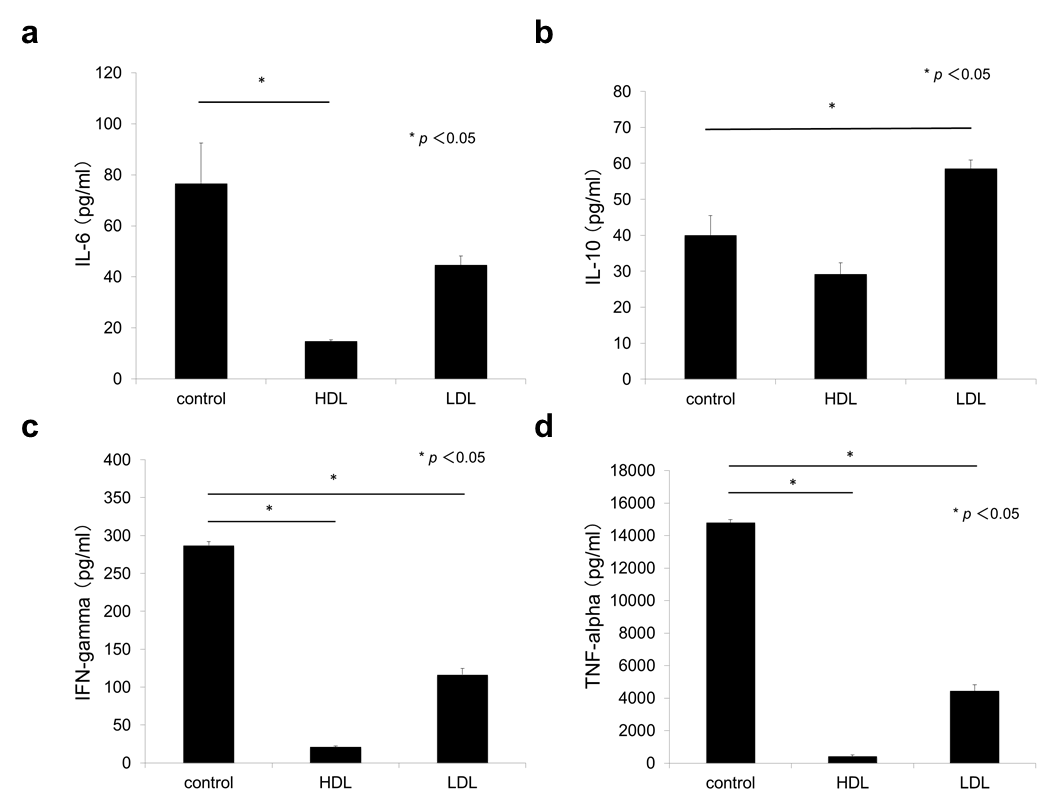
**

**Supplementary Figure S1. Effects of HDL on the production of various cytokines by BCG-infected THP1 macrophages.**

Differentiated THP1-derived macrophages were cultured with or without (control) adding HDL or LDL (50 µg/ml) for 24 hours. The treated macrophages were then infected with BCG (MOI = 10) for 24 hours. The amount of IL-6 (a), IL-10 (b), IFN-gamma (c), and TNF-alpha (d) production from BCG-infected macrophages was measured using the Bio-Plex Multiplex System. ANOVA was used to analyze the data.

**Supplementary Figure S2. HDL suppresses TNF-alpha production of *M. tuberculosis*-infected human macrophages differentiated from blood monocytes.**

The human monocyte differentiated macrophages were cultured with or without (No-infected and Infected control) adding HDL or LDL (50 µg/ml) for 24 hours. The macrophages were then infected with Mtb (multiplicity of infection = 10) for 24 or 48 hours. The amount of TNF-α in the cell culture supernatant was measured by ELISAs. **, *p*-values were less than 0.01 by ANOVA.


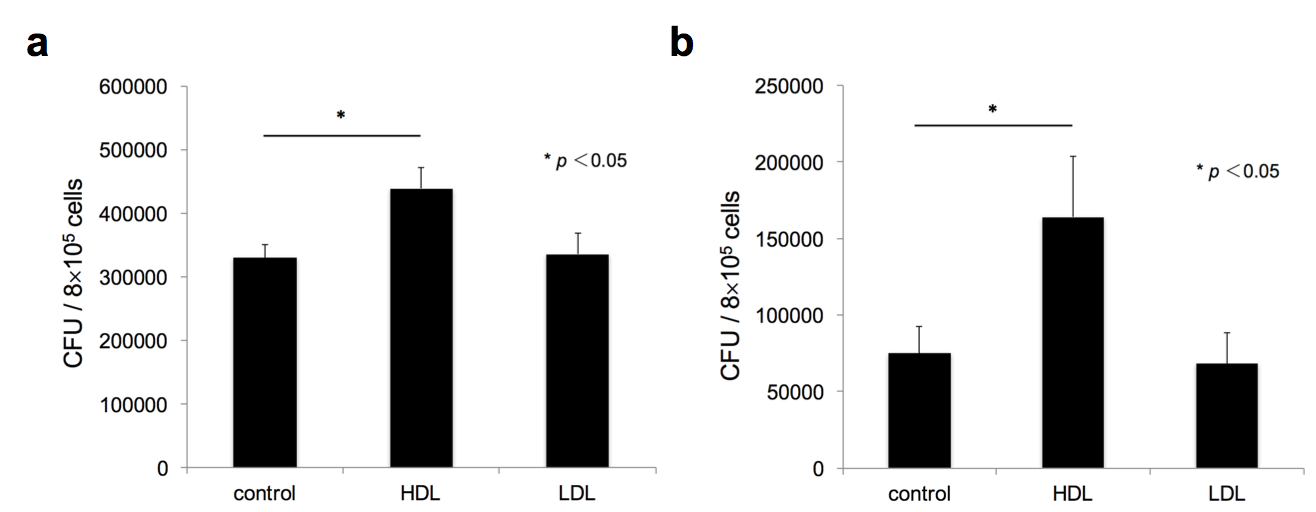


**Supplementary Figure S3. Effects of HDL and LDL on the phagocytosis of *M. tuberculosis* and BCG by macrophages.**

Differentiated THP1-derived macrophages were cultured with or without adding HDL or LDL (50 µg/ml) for 24 hours. The treated macrophages were infected with (a) *M. tuberculosis* (strain H37Rv) or (b) BCG (multiplicity of infection = 10) for 3 hours. The number of colony-forming units (CFUs) resulting from each condition are shown (n=6). These assays were repeated twice. ANOVA was used to analyze the data.


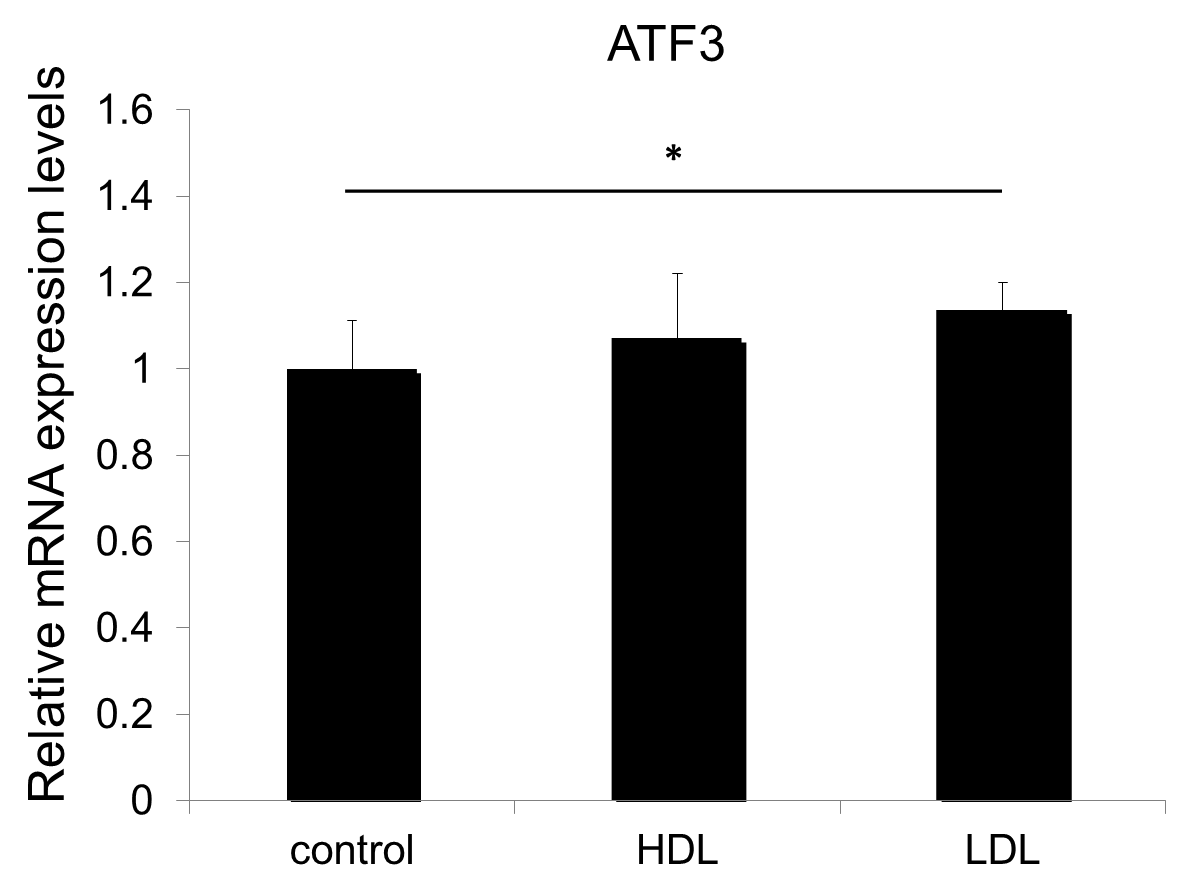


**Supplementary Figure S4. Effects of HDL on the ATF3 mRNA expression level**

Differentiated THP1-derived macrophages were cultured with or without (control) adding HDL or LDL (50 µg/ml) for 6 hours. The ATF3 mRNA expression level in macrophages was then quantified using real-time PCR (n = 3) and normalized to GAPDH mRNA levels. ANOVA was used to analyze the data. This assay was repeated three times.


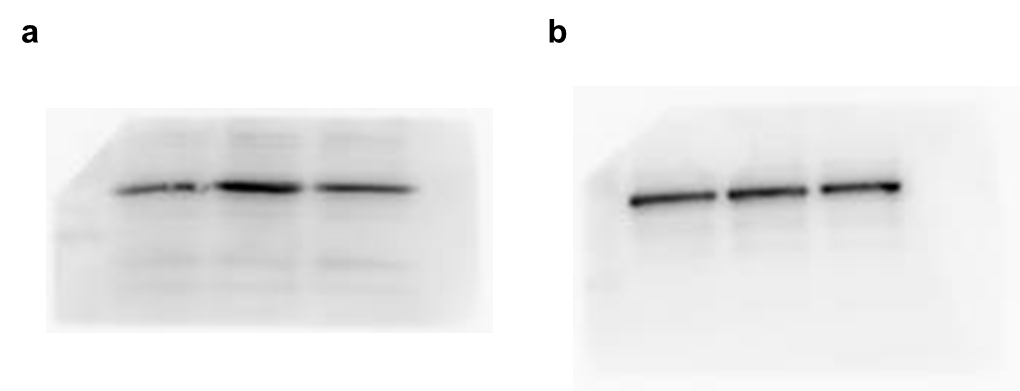


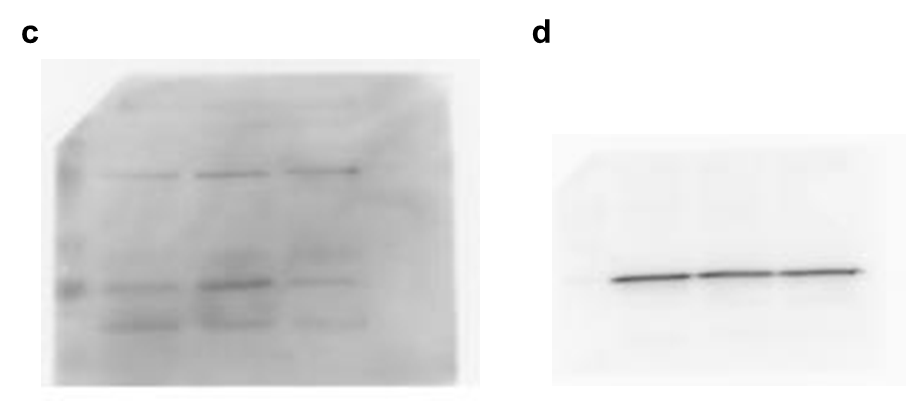


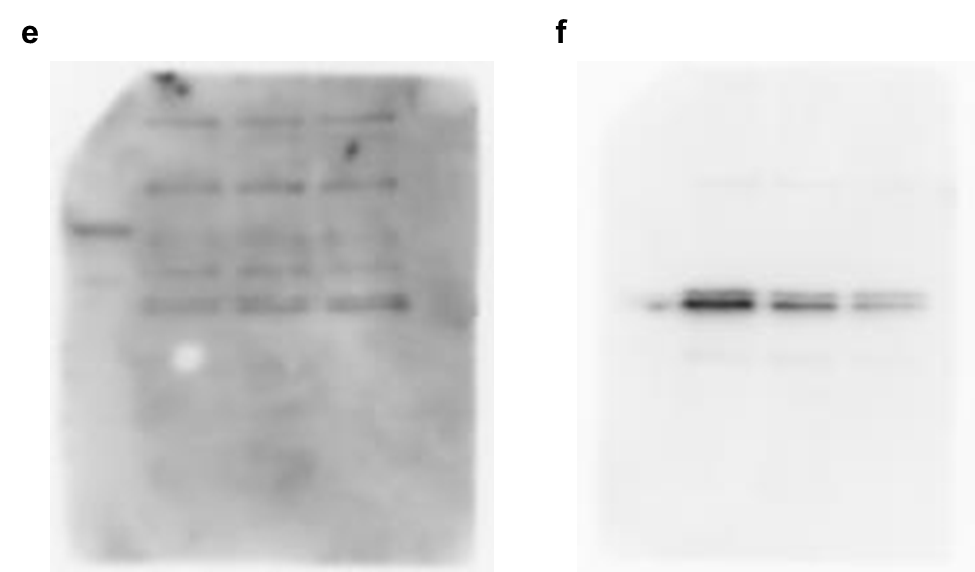


**
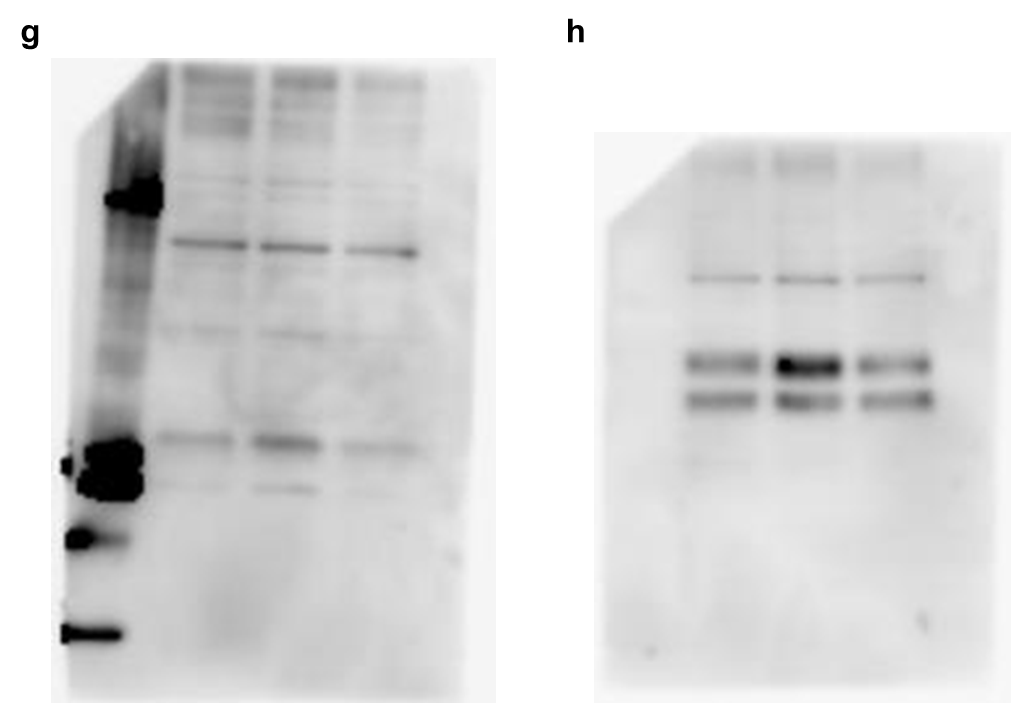

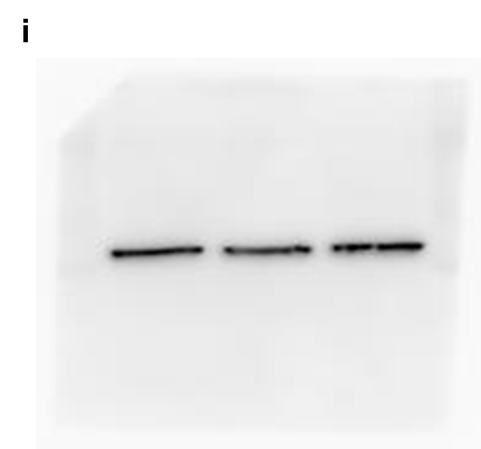
**

**Supplementary Figure S5. HDL impairs activation of TLR2-mediated intracellular signalings.**

Full length western blots of Fig. 4 were presented as Supplementary Fig. S5. Immunoblot of (a) p65 phosphorylation (phospho-p65), (b) total p65, (c) p38 phosphorylation (phospho-p38), (d) total p38, (e) ERK phosphorylation (phospho-ERK), (f) total ERK, (g) JNK phosphorylation (phospho-JNK) and (h) total JNK (relative to (i) total β-actin) were detected. Each whole cell lysates derived from no infection control cells (left), BCG-infected cells (center) and HDL-treated BCG infected cells (right), were fractionated by SDS-PAGE and transferred on a membrane. The membrane c, e, and g include molecular markers on the left lanes. Immunoblot was performed to evaluate each protein level with anti-p65 phosphorylation (phospho-p65), total p65, p38 phosphorylation (phospho-p38), total p38, ERK phosphorylation (phospho-ERK), total ERK, JNK phosphorylation (phospho-JNK), and total JNK (relative to total β-actin), and total beta-actin specific antibodies.


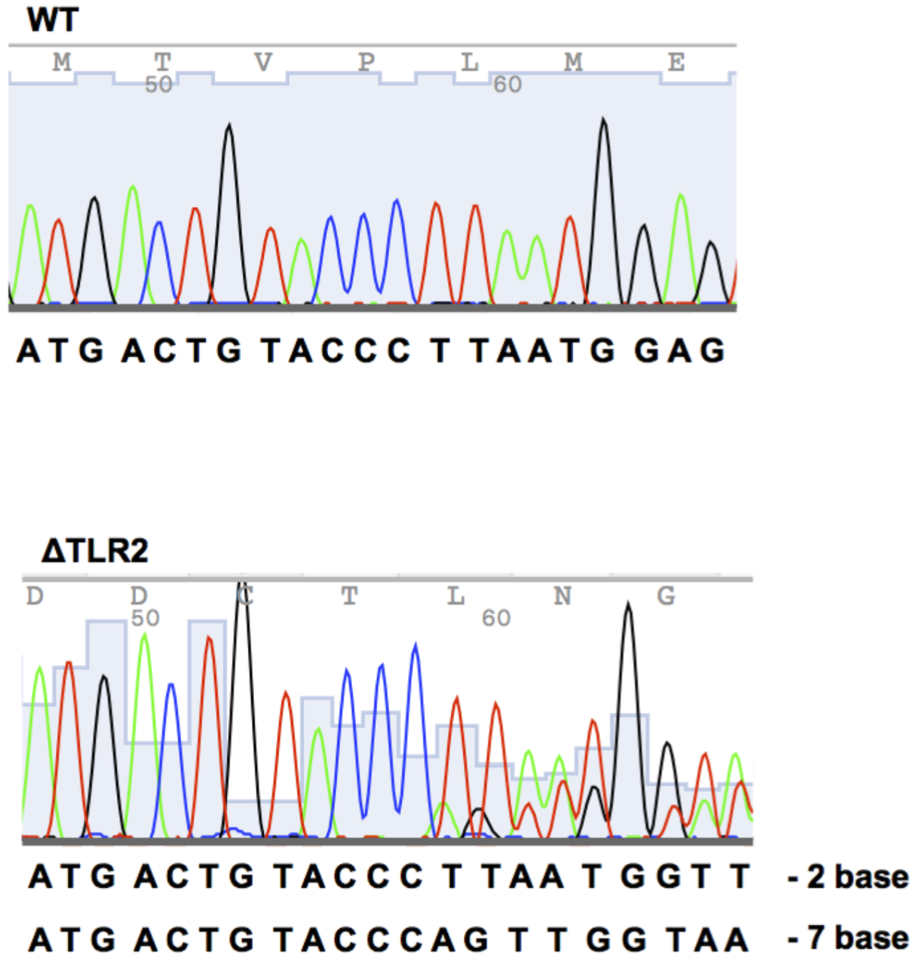


**Supplementary Figure S6. DNA sequence of TLR2 exon of wild-type and TLR2-KO THP1 cells.**

After genomic DNA was purified from wild-type (upside) and TLR2-KO (downside) THP1 cells, targeted region of TLR2 was amplified by PCR and DNA sequence was carried out. Mismatches by deletions with 2 and 7 based in either allele was detected in TLR2-KO THP1 cell.


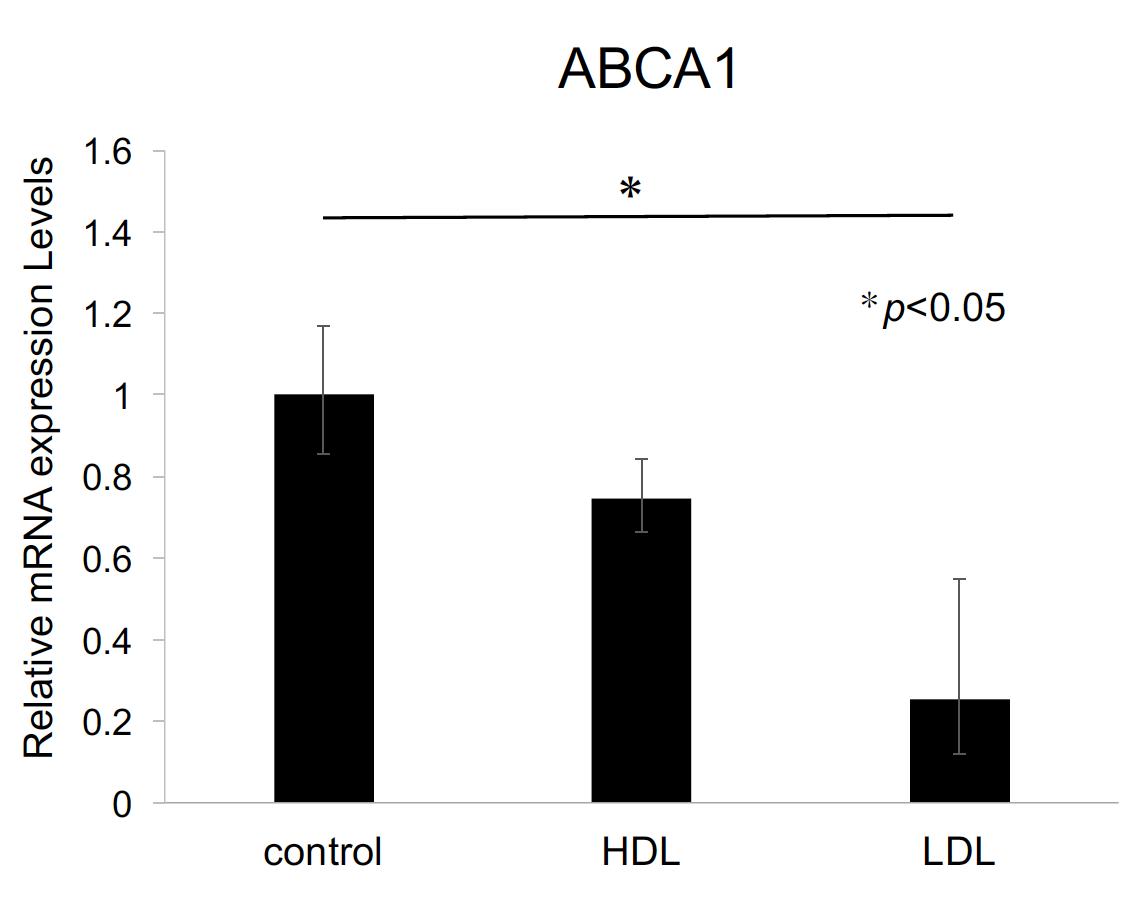


**Supplementary Figure S7. Effects of HDL on the ABCA1 mRNA expression level**

Differentiated THP1-derived macrophages were cultured with or without (control) adding HDL or LDL (50 µg/ml) for 6 hours. The ABCA1 mRNA expression level was then quantified using real-time PCR (n = 3) and normalized to GAPDH mRNA levels. ANOVA was used to analyze the data. This assay was repeated three times.
